# Acute myocarditis following administration of BNT162b2 vaccine

**DOI:** 10.1016/j.idcr.2021.e01197

**Published:** 2021-06-16

**Authors:** Mhd Baraa Habib, Tahseen Hamamyh, Ahmed Elyas, Mohammed Altermanini, Mawahib Elhassan

**Affiliations:** a Internal Medicine Department, Hamad Medical Corporation, Doha, Qatar; b Cardiology Department, Heart Hospital, Hamad Medical Corporation, Doha, Qatar

**Keywords:** COVID-19, coronavirus disease 2019, SARS-CoV-2, severe acute respiratory syndrome coronavirus 2, COVID-19, SARS-COV-2, Myocarditis, BNT162b2, Pfizer-BioNTech, Vaccine

## Abstract

The wide use of vaccines was linked with rare but serious complications in adults, causing a global concern. Here, we describe a young male patient, who presented with acute myocarditis, three days after the second dose of BNT162b2 vaccine.

## Highlights

• To highlight acute myocarditis as a potential adverse complication of BNT162b2 vaccine.
• To illustrate the clinical approach and investigations of myocarditis secondary to BNT162b2 vaccine.
• Conservative management might be reasonable in mild cases.

## Introduction

Myocarditis is an inflammation of the heart muscle, leads to myocyte injury or necrosis. Its presentation ranges from mild sub-clinical course to fatal manifestations such as cardiogenic shock. Myocarditis can be idiopathic in etiology, but the known causes have been classified into infectious, especially viral, autoimmune-related, or as a consequence to toxins exposure, or even vaccinations. The diagnosis of myocarditis usually depends on the combination of the suggestive clinical scenario, elevated cardiac enzymes and cardiac imaging; particularly cardiac MRI (CMRI) [1].

Coronavirus 2019 infection (COVID-19) is a devastating global pandemic, which so far caused a death of over 3 million patients worldwide [2]. Several vaccines have been urgently developed to prevent the occurrence of the disease and to ameliorate its severity. Among these, BNT162b2 (Pfizer-BioNTech) vaccine, which is an mRNA-based vaccine that has been approved by FDA for use in patients above the age of 16, showed excellent efficacy and safety profile. However, the long-term side effects are still under investigation [3].

## Case report

**Fig. 1:**
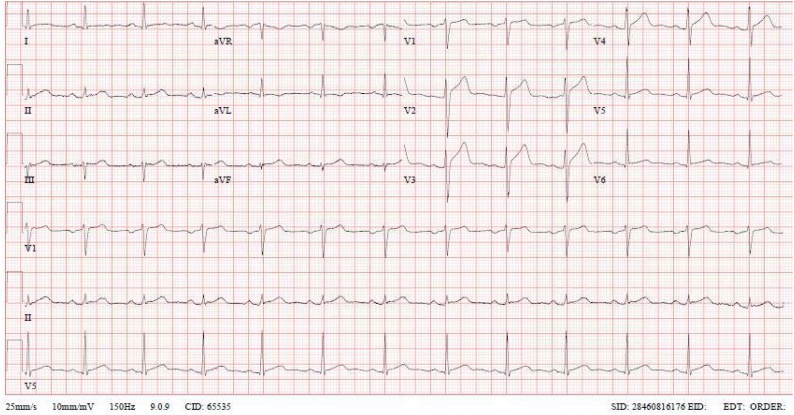
ECG upon admission shows sinus rhythm with mild ST-segment elevation in anterior and inferior leads.

A 37-year-old Filipino man, ex-smoker, drinking alcohol occasionally, was on regular bisoprolol for hypertension, with no history of cardiac disease, presented to the emergency department on 23 April 2021 with a new onset of severe chest pain starting three days after receiving the second dose of BNT162b2 vaccine. The pain was retrosternal, non-radiating, squeezing in nature, with no change to body position or breathing. It was preceded by generalized body aches, fever, chills, and headache for one day, but no other complaints. Upon presentation, his temperature was 36.9 °C, blood pressure 146/86 mmHg and heart rate 98. His oxygen saturation was maintained above 94 %. He had a normal physical examination. Based on the presentation and risk factors, acute coronary syndrome and myo-pericarditis were suspected. Pneumonia was also considered. Electrocardiogram (Fig. 1) showed mild ST-segment elevation in anterior leads. Laboratory tests revealed high level of troponin T (troponin T = 1138 ng\L) with otherwise unremarkable blood work (Table 1). Polymerase chain reaction (PCR) test on a nasal swab was negative for SARS-COV-2, influenza and other common respiratory viruses. Serology tests were negative for Epstein-Barr virus (EBV), Cytomegalovirus (CMV), human immunodeficiency virus (HIV) and viral hepatitis B and C.

**Table 1:** Laboratory tests upon admission.

Detail	Value w/Units	Normal Range
White blood cell count	11.7 × 10^3/uL	4.0−10.0
Hemoglobin	15.5 gm/dL	13.0−17.0
Urea	2.0 mmol/L	2.5−7.8
Creatinine	89 umol/L	62−106
Sodium	139 mmol/L	133−146
Potassium	3.7 mmol/L	3.5−5.3
Bicarbonate	25 mmol/L	22−29
High sensitivity Troponin-T HS	1,138 ng/L	3−15

**Fig. 2:**
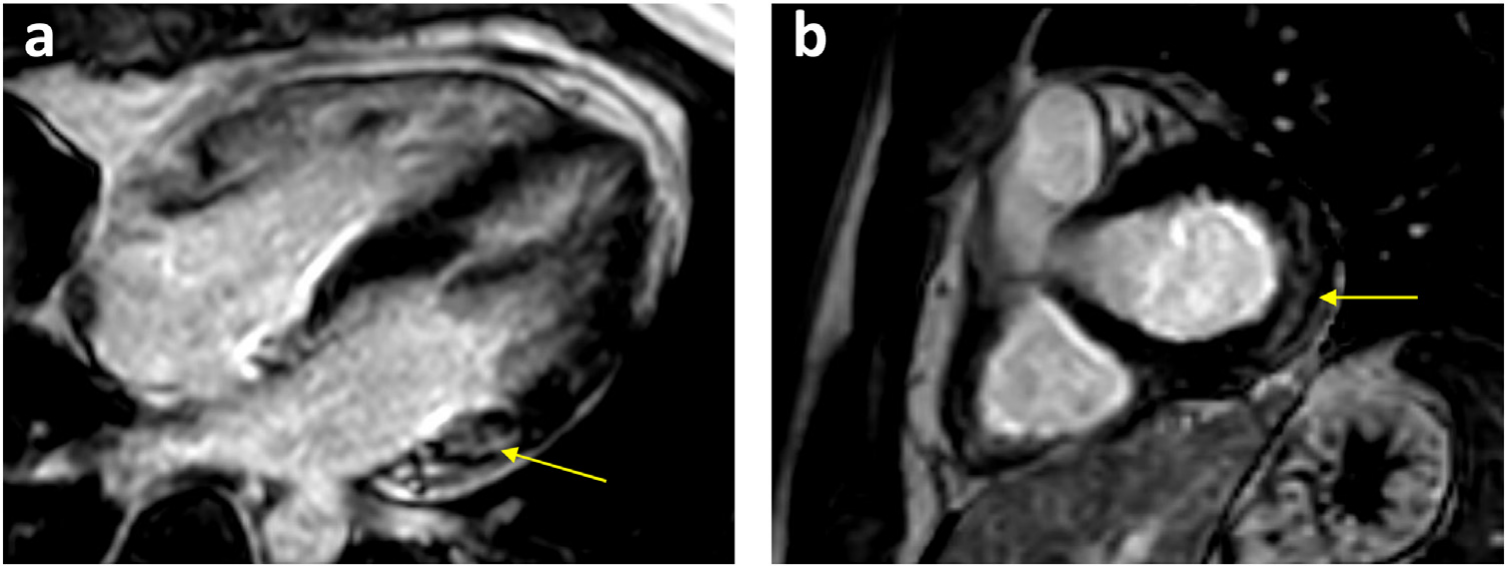
Cardiac MRI PSIR– LGE views showing late gadolinium subepicardial enhancement in basal lateral segments in both (a) Four Chamber and (b) Short Axis views.

Echocardiogram showed normal left ventricular systolic and diastolic function with an ejection fraction (EF = 57 %). There were no regional wall motion abnormalities or pericardial effusion. CT coronary angiography showed no evidence of coronary artery disease with calcium score of zero. The patient then underwent cardiac magnetic resonance imaging (CMRI) which revealed an early and late faint subepicardial enhancement of the basal lateral wall, suggestive of myocarditis (Fig. 2), T2 weighted images showed no clear evidence of myocardial oedema.

**Fig. 3:**
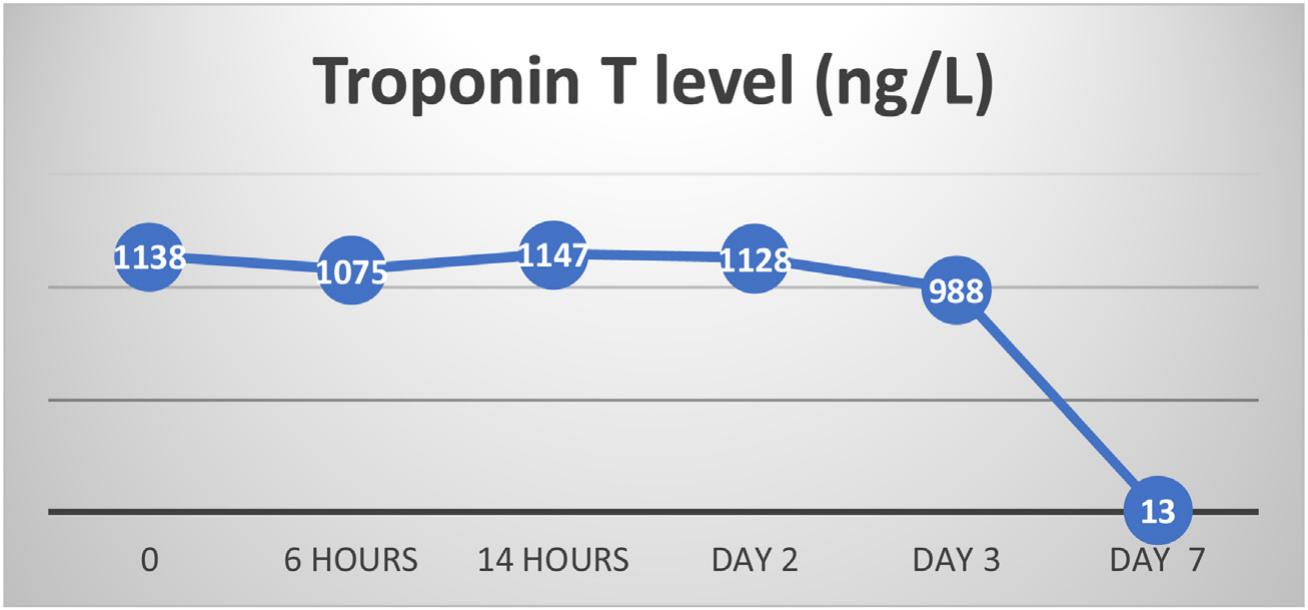
Troponin T level during hospitalization.

The patient was admitted to coronary intensive care unit and started initially on dual antiplatelets, therapeutic anticoagulation and metoprolol for the possibility of acute coronary syndrome. Anti-ischemic medications were held after ruling out coronary artery disease by CT coronary angiogram. At first, he received paracetamol IV for chest pain, then during hospitalization, he became completely asymptomatic, no arrythmias have been recorded, and troponin level was trending down (Fig. 3). He was discharged home after 6 days of hospitalization. The patient was discharged home in an excellent clinical condition. Follow-up phone consultation was after one week of discharge. He was doing well and denied any active complaints.

## Discussion

Acute cardiovascular events including myocarditis were reported in the setting of COVID-19 infection [4], but the underlying mechanism is not well understood and might be related to an autoimmune reaction or a cross-reactivity between SARS-CoV2 antigens and myocardial cell proteins [5]. Several vaccines were developed against SARS-CoV2 and are being used worldwide. Rare but serious thrombotic events were reported after ChAdOx1 nCov-19, AstraZeneca vaccine administration in young females, raising a global concern [6]. In BNT162b2 vaccine trial, the common adverse events were generally mild including pain at the injection site, fever, fatigue, and headache. Although no deaths were attributed to the vaccine, one patient died from cardiac arrest and there was one case of paroxysmal ventricular tachycardia in the vaccine arm (3).

Two case reports have been published in the medical literature about myocarditis post BNT162b2 vaccination [7,8]. Like our case, Ammirati et al. reported a male patient who had myocarditis 72 h after the second dose of BNT162b2 vaccine with favorable hospital course (8). In contrast to our patient, he had a previous COVID-19 infection, and his Troponin T levels were lower. Taking into account the acute history, the short interval between the administration of vaccination and the presentation of myocarditis, and the exclusion of other common causes, BNT162b2 vaccine-induced myocarditis is the most likely diagnosis in our patient.

Myocarditis was previously reported after immunization in relation to several vaccines, especially smallpox vaccine [9]. The mechanism is unknown, but it is thought to be related to immunological response following vaccination due to molecular similarities between the vaccine and cardiac cell proteins or secondary to vaccination nonspecific inflammatory response [10]. It is noteworthy that myocarditis as a complication might be under reported if the patient had transient symptoms and did not seek medical advice or underwent limited investigations.

## Conclusion

Although COVID-19 vaccination may prevent the infection and its severe complications in most of cases, serious side effects should be considered. In this case, we would like to highlight acute myocarditis as an important adverse effect of BNT162b2 vaccine especially in young males after receiving the second dose of the vaccine. We think this association needs to be explored in large studies and needs to be considered in patients who experience cardiac symptoms post BNT162b2 vaccine administration.

## Funding

This research did not receive any specific grant from funding agencies in the public, commercial, or not-for-profit sectors.

## Ethical approval

Ethical Approval was obtained by Medical Research Center (MRC) under ID MRC-04-21-416 on 03/05/2021.

## Consent

Written consent was obtained from the patient to publish this case.

## CRediT authorship contribution statement

**Mhd Baraa Habib** have written the initial draft of the manuscript. **Tahseen Hamamyh:** participated in literature review and editing the manuscript. **Mohammed Altermanini:** literature review. **Ahmed Elyas** and **Mawahib Elhassan:** editing, revision and mentorship.

**Mhd Baraa Habib**, **Tahseen Hamamyh**, **Ahmed Elyas**, **Mohammed Altermanini** and **Mawahib Elhassan** critically reviewed the initial and the final draft of the manuscript and approved it for submission.

## Declaration of Competing Interest

The authors report no declarations of interest.
